# Povidone-Iodine as an Adjuvant Therapy for Refractory Gonorrhea Keratoconjunctivitis: A Case Report

**DOI:** 10.7759/cureus.83676

**Published:** 2025-05-07

**Authors:** Sinan Albear, Stephen LoBue, Ayorinde Cooley, Traeson Brandenburg, Rebecca Friedes, Jennifer Park

**Affiliations:** 1 Ophthalmology, State University of New York Downstate Medical Center, Brooklyn, USA; 2 Ophthalmology, Willis-Knighton Medical Center, Shreveport, USA

**Keywords:** gonorrhea conjunctivitis, keratoconjunctivitis, neisseria gonorrhoeae, povidone-iodine, refractory

## Abstract

Gonorrhea keratoconjunctivitis is a sight-threatening condition that requires hospitalization and systemic antibiotic therapy. We report a case of a 47-year-old man suffering from gonococcal conjunctivitis with progressive corneal involvement despite three days of appropriate topical and intravenous antibacterial therapy. The patient received adjuvant treatment with 5% topical povidone-iodine, which had an immediate beneficial effect. One day after the application, the patient showed a resolution of severe exudative discharge with improvement in epithelial defects and thinning. By hospital day seven, the corneal defect and thinning had both resolved. Povidone-iodine may be a low-cost, safe, and potentially effective adjuvant therapy for refractory gonococcal keratoconjunctivitis in adult individuals unresponsive to standard-of-care antibiotic therapy after 72 hours of treatment. However, there is no evidence to support povidone-iodine as a standalone treatment for gonococcal conjunctivitis. Further research with a greater number of patients is needed to determine its true utility.

## Introduction

Gonococcal conjunctivitis is typically thought of as a neonatal infection (ophthalmia neonatorum), but it has shown increasing prevalence among the adult population [1]. Prompt diagnosis and treatment of gonococcal keratoconjunctivitis is crucial, as delay in treatment can lead to significant scarring and corneal perforation [2]. With early diagnosis and intervention, most patients respond to treatment within 24-48 hours after antibiotic therapy [2]. Resistance to the currently recommended therapy for gonococcal conjunctivitis (ceftriaxone and azithromycin) is of global concern; therefore, there is a need for new and effective treatments [3]. Povidone-iodine is an effective antimicrobial agent against a variety of organisms, including bacteria, viruses, fungi, and protozoa [4]. Several aspects of povidone-iodine are appealing, including low cost, rare microbial resistance, and no cross-resistance to antibiotics [5].

Presently, povidone-iodine is widely used as an effective method of eradicating surface microorganisms prior to intraocular surgery. It is also used by eye banks during the corneal tissue recovery process to decrease the incidence of post-keratoplasty infections [6]. Given its acceptability and effectiveness in prophylaxis and treatment of various ophthalmologic infections, we utilized povidone-iodine in treating a refractory adult gonococcal conjunctivitis.

## Case presentation

A 47-year-old man with a medical history of coronary artery disease, hypertension, and hyperlipidemia presented to the emergency department with bilateral (OU) eye discharge and hyperemia for three days. The patient went to an urgent care center three days prior and was started on polymyxin B sulfate and trimethoprim (polytrim) eye drops with no improvement.

The patient denied any contact with individuals with eye redness or discharge. He denied a history of sexually transmitted illnesses (STI). However, the patient had multiple sexual encounters with women and reported using barrier protection inconsistently. He denied smoking, alcohol, or illicit drug use.

**Figure 1:**
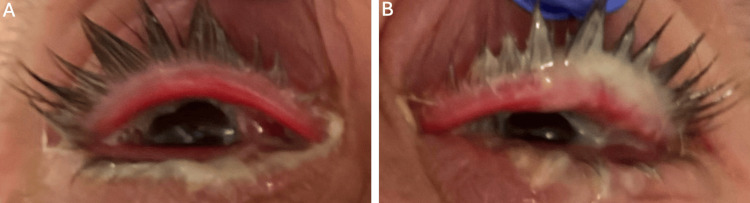
External exam noting copious purulent discharge with mild swelling and hyperemic lid margins of the A) right eye (OD) and B) left eye (OS).

On day one of our examination, Snellen visual acuity was 20/200 in the right eye (OD) and 20/400 in the left eye (OS). Intraocular pressure was 16 mmHg OD and 17 mmHg OS. On external exam, copious purulent discharge and non-tender palpable preauricular lymph nodes were noted bilaterally (Figure 1).

**Figure 2:**
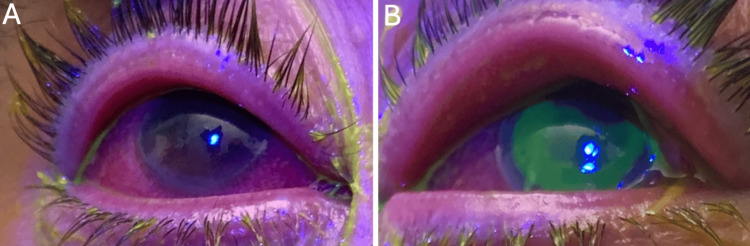
The slit-lamp examination was remarkable for 4+ bulbar and palpebral injection with conjunctival papillae in both eyes (OU). A) Diffuse superficial punctate keratitis (SPK) was noted in the right eye (OD). B) A large epithelial defect, 10x10 mm, was noted in the left eye (OS).

An external eye exam was notable for mild swelling and hyperemic lid margins bilaterally. Slit-lamp examination was remarkable for 4+ bulbar and palpebral injection OU, tarsal conjunctival papillae OU, diffuse superficial punctate keratitis (SPK) OD, and a large epithelial defect OS (Figure 2).

**Figure 3:**
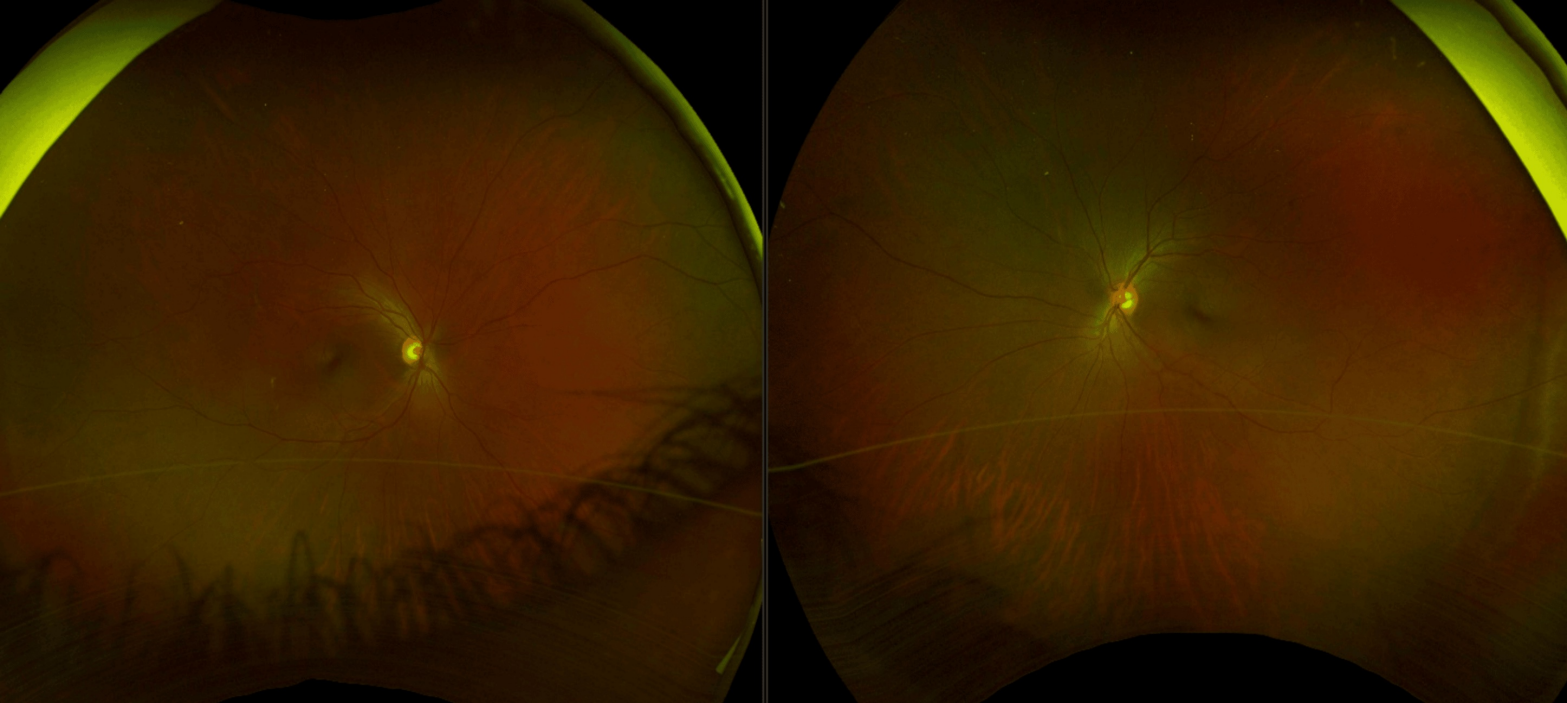
Representative fundus photo. Ultra-widefield retinal imaging depicts the fundus finding seen on clinical exam. A clear media was noted without the presence of vitritis in both eyes. The optic nerve was well perfused with a cup-to-disc ratio of 0.4. Normal caliber and course of the vessels without vasculitis were documented. The macula and surrounding retina were clear without any signs of retinal or choroidal lesions in either eye.

No anterior chamber inflammation was noted. Both eyes had a normal fundus exam without vitritis or macular lesions (Figure 3).

His vital signs were within normal limits. The patient was hospitalized and started on intravenous (IV) ceftriaxone 1 g every 12 hours, 1 g azithromycin po, moxifloxacin drops every hour, and erythromycin ointment three times a day. On day three of presentation, his epithelial defect OS remained unchanged, and 30% peripheral corneal thinning developed OS. The patient continued to exhibit mucopurulent discharge OU. A bacterial culture of the purulent conjunctival discharge subsequently returned positive for gonococcal gonorrhea. Additional testing for HIV and syphilis was negative.

**Figure 4:**
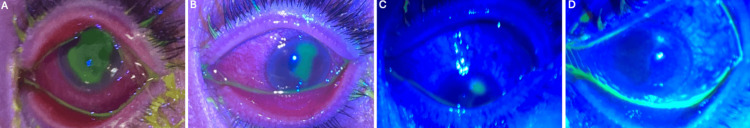
On admission day three, 5% povidone-iodine solution was placed in the eye for one minute, followed by a saline lavage to thoroughly flush the eye. A) Twenty-four hours after povidone-iodine treatment, his ocular discharge resolved bilaterally, and his corneal defect and thinning had improved by roughly 10% (stained in green). B) Two days after povidone-iodine treatment, corneal defect and thinning improved 60%, C) which improved 90% three days after initial treatment. D) By day seven of presentation or four days after povidone-iodine treatment, the epithelial defect and corneal thinning completely resolved.

Five percent ophthalmic povidone-iodine drops were instilled into both eyes, including the conjunctival fornices. His eyelashes were scrubbed with cotton swabs soaked in 5% ophthalmic povidone-iodine. The solution was left in place for one minute, and a saline lavage was subsequently used to thoroughly flush the eye. The next day, his ocular discharge resolved bilaterally, and his corneal defect and thinning had improved by roughly 10% (Figure 4A).

Two days after povidone-iodine treatment, the corneal defect and thinning improved by 60% (Figure 4B). By day seven of the presentation, the epithelial defect completely healed, corneal thinning resolved, and visual acuity improved to 20/30 OD and 20/50 OS (Figure 4D). IV ceftriaxone, po azithromycin, and topical moxifloxacin and erythromycin were stopped after seven days of treatment.

The patient was educated on safe sexual behavior. The patient was also asked to discuss his STI status with his partners so they could receive appropriate treatment.

## Discussion

Gonococcal keratoconjunctivitis characteristically manifests abruptly with copious purulent discharge with corneal involvement [7]. Corneal findings are highly variable and may include corneal edema, subepithelial or stromal infiltrates, marginal corneal melt, and ulceration, which can progress to full-thickness corneal perforation [8].

Our patient was a 47-year-old man with a history of unprotected sexual intercourse with multiple partners who developed mucopurulent discharge bilaterally, unresponsive to polytrim eye drops. On exam, he was noted to have copious purulent discharge, severe conjunctival injection, diffuse SPK OD, and a large epithelial defect OS, consistent with a diagnosis of gonorrhea keratoconjunctivitis (later confirmed via conjunctival culture). Given corneal involvement, he was started on IV ceftriaxone and azithromycin, as well as moxifloxacin Q1h and erythromycin ointment TID. After three days of the aforementioned treatment, he continued to have significant mucopurulent discharge and a non-healing corneal defect. Ophthalmic povidone-iodine 5% drops were instilled at this time, and by the following day, discharge resolved bilaterally along with improvement in his corneal epithelial defect and thinning. After another three days (hospital day seven), the corneal defect and thinning had both resolved.

Several studies have shown the efficacy of povidone-iodine in disinfecting against Neisseria gonorrhoeae, the causal organism of gonococcal conjunctivitis. The mechanism of action involves the release of free iodine, which disrupts critical cellular processes including cell membranes, enzymes, and nucleic acid structure of the bacteria [5,9]. In vivo studies have focused mainly on the prophylaxis of ophthalmia neonatorum [10,11].

Povidone-iodine has proven useful in disinfecting against microorganisms, including Neisseria gonorrhoeae, and treating various forms of keratitis and conjunctivitis. Thus, we believed that adding it to our patient’s treatment regimen helped eradicate the infection as significant symptoms persisted and even worsened despite being on the gold-standard regimen for 72 hours [12]. Improvements noted one day after the application of povidone-iodine drops suggest that it may have a significant beneficial effect on severe gonococcal conjunctivitis.

The safety profile and efficacy of 5% povidone-iodine have been extensively studied in the literature and successfully applied to a range of periocular and ocular sites such as the eyelids, cornea, and conjunctiva [13,14]. Furthermore, hypersensitivity reactions to povidone-iodine are exceedingly rare. Even in patients with a history of allergy to iodine-containing substances such as shellfish or contrast media, there is little evidence of cross-reactivity to povidone-iodine. There are currently no reports of anaphylaxis to topical ophthalmic use of povidone-iodine [5].

Nevertheless, povidone-iodine has been associated with mild ocular irritation, including transient burning sensations, superficial keratitis, and conjunctival injection. Povidone-iodine should always be thoroughly washed out after application and never combined in an alcohol based formula, which can further exacerbate ocular irritation. Its general lack of adverse effects, along with its low cost and little to no resistance, has allowed it to be widely accepted. Thus, we considered the potential benefit of using povidone-iodine to outweigh any risks.

The optimal concentration and exposure timing of povidone-iodine are highly contentious. Some studies have demonstrated possible damaging effects of povidone-iodine on the corneal endothelium and epithelium, which is highly conflicting and likely due to varying concentrations and time of exposure [9,10].

As a result, significant debate has been involved in the appropriate concentration of povidone-iodine solutions. Some studies suggest that more dilute povidone-iodine solutions (such as 0.1%) have greater bactericidal activity, attributable to an increase in free iodine [15,16]. On the other hand, other studies have shown 5% povidone-iodine to be more effective in reducing bacterial load. The current guidelines from the American Academy of Ophthalmology and the European Society of Cataract and Refractive Surgeons recommend 5% povidone-iodine use prior to any surgery as higher concentrations can induce more irritation, injection, and corneal edema [13,14]. Thus, we used a 5% povidone-iodine solution for our patient. More dilute or more concentrated solutions may have different outcomes and is worthy of future investigation.

## Conclusions

Utilizing 5% povidone-iodine may be a potentially effective adjuvant therapy for refractory gonococcal keratoconjunctivitis in adult individuals unresponsive to standard-of-care antibiotic therapy after 72 hours. Its low cost, broad-spectrum efficacy, and minor side effects make povidone-iodine a highly desirable tool worldwide. However, there is no evidence to support povidone-iodine as a standalone treatment for gonococcal keratoconjunctivitis. Further research with a greater number of patients is needed to determine its true utility.
